# Supplementary material for: Contextualizing sacrificial dilemmas within Covid-19 for the study of moral judgment
Source: PLoS One. 2022 Aug 22;17(8):e0273521. doi: 10.1371/journal.pone.0273521 (PMC9394814; doi:10.1371/journal.pone.0273521)
Supplement: Attachment — Submitted filename: RESPONSE.Reviewers.docx [file pone.0273521.s002.docx]

Response to reviewers

Please find below the reviewers’ comments and the corresponding responses. We pay attention to make the changes easy to track in the revised manuscript file: the blue ink is used to make salient all changes, whereas the red ink is used to signal the passages to suppress in the final version.

We would like to thank the two reviewers for their helpful comments that enabled us to improve the first version of the manuscript.

We hope all responses provided and all changes reported in the manuscript will correspond to their expectation.

Reviewer #1: The experiments conducted are very interesting but I have some doubts about their methodology and purported realism, which is very important given that that’s the topic under investigation.

*Lengthy introduction gives useful introduction to the literature in this area. However, there* ***are a few shortcomings, mainly from the ethical perspective:***

*The language used here presents utilitarianism as unethical/an opponent of ethics. “From an ethical point of view” should say “from a deontological” point of view, or some other moral system. “From a utilitarian point of view, approving the sacrifice of one life in order to save the lives of five others is morally acceptable with respect to the number of lives saved. By contrast, from an ethical point of view, it is not acceptable to sacrifice the life of one person in order to save others because the value of each human life is paramount and no one has the right to sacrifice a life.”*

**Answer**: We agree with you that the utilitarian principles should not be presented as unethical/ opposite to ethics. Indeed, a utilitarian would consider that maximizing lives saved is the moral and ethical thing to do. To take your comment into account, we modified the following passage:

**Line 45 to line 50**. “From a utilitarian point of view, approving the sacrifice of one life in order to save the lives of five others is morally acceptable with respect to the number of lives saved. By contrast, from a deontological point of view, it is not acceptable to sacrifice the life of one person in order to save others because the value of each human life is paramount and no one has the right to sacrifice a life, regardless of any benefits that may arise in doing so.”

**Please see also Line 59 and Line 118.**

*It's not the “footbridge dilemma”, it’s the most famous variation of the trolley problem. And “Someone is standing on the tracks of a bridge” doesn’t make sense.*

*Why use the fat man example rather than the classic trolley problem? Using the fat man example instead makes it sound like you intended people to die when you deny them an intensive care bed, whereas that’s not the case.*

**Answer:** Taking into account your comments, we revised the corresponding passage to present the classic trolley problem (Foot, 1967; Thomson, 1976). **Line 52 to 56.**

*More argumentation is needed to back up the claim that “emotion makes utilitarian decisions harder to make”. For* ***many people the utilitarian choice is justified by an appeal to emotion.*** *Also, people might intuitively believe that they should act deontologically, but later on reflection regret not making the utilitarian decision*.

**Answer:** To be honest, your comment invited us to consider a more balanced perspective on moral judgment explanation. Additional references helped us to introduce another theory (intuitionist theory) which is an additional relevant explanation of how moral judgment is expressed. Thus, we revised the introduction accordingly (**line 64 to 86**) so that the conflict between the utilitarian and deontological responses is now approached through alternative models. By doing this, we provided the readers with dominant models of moral judgment: the dual-process model (Greene et al. 2001, 2004, 2008) and the intuitionist models (Haidt, 2001; Dubljević & Racine, 2014, 2017; see also Dubljević et al., 2018; Dubljević, 2020).

*It’s true that the footbridge dilemma is unrealistic. However, the main trolley problem is much more realistic and that’s another reason it should be used in this paper instead.*

**Answer**: We agree with you, so that we revised the corresponding passage to present the trolley problem. Please, see **line 52 to 56**.

*Again, the “switch dilemma” isn’t “a version of the trolley problem”, it IS the trolley problem.*

**Answer**: We agree with you, so we revised the corresponding passage as following: “Navarrete et al. were the first to implant, in immersive VR, the trolley problem consisting in switching the trolley’s direction to kill one person instead of five” **Line 140 to 141**.

*One drawback of the VR approach is that it risks making the experiment seem more like a game than an abstract scenario.*

**Answer**: To introduce your idea that the increase of utilitarian responses in virtual moral dilemmas could be a result of artificial gaming behaviors, we inserted a passage in **line 153 to 158.**

“This difference could be explained by the fact that the VR format of the dilemmas could elicit artificial gaming behaviors where sacrificial action is trivialized. Francis et al. (2017) attempted to exclude this possibility in their work by taking into account individuals' previous gaming experience and its influence on moral decision making. Although no such link was established in their studies, this drawback of VR format cannot be discarded for sure”.

*I’m not the greatest fan of trolley problems, but it’s overstating it to say ““trolley problems are unrealistic and unlike anything people encounter in the real world”. Anyone who has swerved the car to avoid a squirrel or fox, putting their family at risk, has experienced a real-life trolley problem. And as you argue in the paper, doctors on the ICU face similar issues.*

*Doctors have always been making ‘moral decisions about which patients to admit to the Intensive Care Unit (ICU) and for which ones to provide lifesaving resources” regardless of the pandemic.*

**Answer**: We agree with your comment but we would like to draw your attention to the fact that we reported a citation from Bauman et al. (2014, p.545) to highlight the gap between classical sacrificial dilemmas (i.e., text or VR format) and those using different contexts (e.g., war, donation, Covid-19 crisis) to increase mundane realism while studying moral decision making.

*ICU sacrifical dilemmas might seem more realistic than trolley problems, but they’re often greatly oversimplified in a way that makes them clinically unrealistic, even if they seem real to members of the public.*

**Answer**: In the Covid-19 pandemic context, intensive care unit sacrificial dilemmas, precisely because they correspond to real-life situations, have already been used in the literature on moral judgment to compensate for the lack of realism of hypothetical dilemmas (e.g., Hugan et al., 2021; Kneer & Hannikainen, 2021; Wilkinson et al., 2020).

However, we agree with your comment that these dilemmas are simplified and may appear clinically unrealistic to the subjects. As we mentioned in the manuscript (line 247 to 282), the argument that doctors may have been led to make a decision, in the Covid-19 pandemic context, whether to sacrifice a one patient to save more lives is not sufficient to assert that dilemmas involving ICU triage are perceived as realistic. Kneer and Hannikainen (2021), for example, argued that sacrificial dilemmas related to the Covid-19 pandemic are high in realism compared to dilemmas unrelated to Covid-19 but they did not measure the perceived realism of the two types of dilemmas. Based on this weakness in previous research (i.e., the lack of measurement of the perceived realism of the scenarios), one of the main purposes of the two present studies was to measure different dimensions of perceived realism (i.e., plausibility, factuality and typicality) in two versions of the triage dilemma: a version contextualized within the Covid-19 pandemic (the supposedly realistic version) and another version not contextualized within the Covid-19 pandemic (the supposedly less realistic one). The two versions of the TRIAGE dilemma differed on the three dimensions of perceived realism: the results showed that the former was perceived as more realistic than the latter.

*The footbridge dilemmas is also implausibly because it seems unlikely the decision-maker could life/shove such a heavy person, and also because there would be no way of knowing whether he would stop the train or not. In contrast, the main trolley problem is much more plausible as it lacks these “stupid” features.*

**Answer**: Indeed, the difference of moral responses between the trolley problem (an impersonal dilemma) and the footbridge problem (a personal dilemma) can be explained by level of certainty about the outcome of the sacrificial action. In line with this interpretation, Shou et al. (2020) showed that participants generally had greater certainty that the five people would survive if they decided to kill the individual in impersonal dilemmas compared to personal dilemmas. Regarding the respective level of certainty about the outcome of the action in these two types of dilemmas, it is not surprising that participants are more utilitarian in the former than in the latter. This additional result, which invites us to consider the plausibility of sacrificial action in the study of moral judgment, was added in the modified version of the manuscript (please see **line 315 to 324**).

In general, the plausibility of utilitarian action has been underestimated in research on moral judgment and, in Study 2 reported in this paper, we investigated the role of this factor in moral responses. In line with previous works (e.g., Körner et al., 2019; Shou & Song, 2017), our results showed that participants were more inclined to choose the utilitarian action in plausible than in implausible versions of the TRIAGE dilemma. Note that the fact that plausibility of action did not have a significant influence on participants’ moral judgment sheds light on the necessity of distinguishing judgment and choice of action in moral responses.

*Why were those who had lost a relative to Covid excluded after participation rather than before, if the criterion was decided before collection? That means that their time was wasted. An ethics committee would have picked up on this if the study had been reviewed.*

**Answer:** It was not possible to exclude participants on this respect at the beginning of the experiment, since asking them a question about the death of a relative due to Covid-19 introduces a bias: making salient the covid-19 pandemic context could have interfered with the context variable we manipulated in this study (dilemmas contextualized and not contextualized within Covid-19).

Participants who were finally excluded from the sample were not informed that their contribution to this study was not taken into account due to this precaution.

*The triage dilemma used in study 1 is unrealistic, at least compared to the ICU bed example. Hardly any hospitals ran out of oxygen and none that I’m aware of ran out of antibiotics. Given the emphasis on plausibility this is unfortunate. The oxygen example also differs from antibiotics (and the ICU bed) because it’s normaly certain that a patient will die without oxygen but that’s not the case for the other two. In addition, the antibiotics example seems implausible because five patients would need five times as much antibiotics as one patient, and antibiotics courses need to be taken for days. To me the oxygen scenario is less plausible than the ICU bed one, but the antibiotic one is deeply implausible which is a major issue given that it was used only in the example that’s supposed to be less plausible for the reason that it’s decontextualised. This highlights that it seems to be a methodological flaw to have oxygen in the contextualised example, but antibiotics in the noncontextualised one? That introduces an unnecessary variable that could act as a confounder.*

**Answer**: If we had used the oxygen issue in both situations, in the non-contextualized dilemma, the participants would have made the connection with the health crisis. This is why we constructed two dilemmas involving two different issues.

However, in these dilemmas, the consequences but also the way of solving the dilemma were the same: taking the element that keeps a person alive to give it to 5 other people. If at the end it is stated that with the taken element the other 5 people will be saved, the participants will automatically make the inference that the taken element makes it possible to save the 5 people.

In both cases, one can imagine the participant thinking that the "oxygen tank/antibiotic dose" taken will not be shared by all 5 patients. This is true, but it is true in both cases. It was this lack of plausibility of the resolution action in Study 1 that prompted us to conduct Study 2. In our view, in both versions of the dilemma, there is indeed a "plausibility of the resolution action" problem, but it is unique to the classic dilemma format. You say that the non-contextualized dilemma is less realistic, because it is more inconsistent; we think that they are both equally inconsistent (because of the resolution action). This is what prompted us to look at the plausibility of the action in Study 2.

In addition, based on the results reported in study 1, we obtained evidence that the two versions of our TRIAGE dilemma were only distinguished according to perceived realism (**Line 419 to 425**) and did not differ as to the plausibility of the stated consequences, which were considered moderately plausible in both versions (**Line 459 to 464**). We agree with you that is important to signal to the readers that, in Study 1, our material could be criticized regarding the plausibility of the stated consequences but not on the perceived realism, on the basis of the data collected.

Consistent with your comment, in the discussion section of Study 1 we already pointed out that it is important to control not only scenario realism but also action plausibility in sacrificial dilemmas to study their respective influence on moral responses (please see **line 476 to 497**).

*Also, looking at the examples it’s clear that they’re both contextualised; it’s just that one is a fictional and one a real-world context.*

**Answer**: Indeed, one version is contextualized within the Covid-19 crisis and can be considered a real-world context (please see line 232 to 235); by contrast, the other one is not contextualized within Covid-19 crisis and could be perceived as fictional like most of the hypothetical scenarios usually used to study moral judgment. In our view, the way we labelled the two modalities of the variable is not opposite to your point of view.

*“Participants were asked to be as honest as possible in their responses, knowing that there was no right or wrong answer.” Were participants told that there is no right or wrong answer in ethics? That’s a very contentious morally relativistic statement that many ethicists would disagree with.*

**Answer**: Indeed, in the ethical puzzle, it would be wrong to say that there is no right or wrong moral response just as it would be wrong to assert that a judgment or action is morally right or wrong.

We emphasized to participants that “there was no right or wrong answer”, not so that they would feel pressured or forced to give one moral response over another, but so that they would “be as honest as possible” in their responses to the dilemmas.

Note that this instruction corresponds to the one usually used in studies on moral judgment (e.g., Watkins & Laham, 2018; Brigaud & Blanc, 2021).

*The results section is very short and could use more textual description of the results.
Also, it would probably make sense to describe the Methods of both studies in one section, the Results in another, and then have a combined discussion.*

**Answer:** The way we presented the two studies corresponds to what we did. Indeed, the two studies were not conducted simultaneously but successively, with the objective, in Study 2, of replicating the results obtained in Study 1 (i.e., no significant effect of perceived realism on moral responses), and of furthering the question of the impact of the plausibility of utilitarian action.

In our view, there are two advantages in presenting the two studies separately: to respect the real context in which the two studies were conducted, and to attempt to replicate results to overcome the reproducibility crisis in scientific studies.

Nevertheless, your comment drew our attention to the fact that the material section of Study 2 was too long and redundant. Therefore, we have presented in this section only the two new versions of the Triage dilemma (i.e., the versions containing information about the plausibility of sacrificial action) since the other two versions (the ones without such information) were identical to those in Study 1. Thus, we reduced the material section of Study 2 while providing enough detail to allow qualified investigators to fully replicate our study.

Regarding the results section of Study 1, as you point out, we have presented the findings concisely, following PlosOne submission guidelines (“We encourage you to present and discuss your findings concisely”).

To the extent that results of Study 1 are replicated in Study 2, we have included these results in the discussion section to explain how they relate to the hypotheses presented and to previous related studies.

Note that in the revised version of the manuscript, we extended the discussion of the results since our data could be interpreted not only in support of the dual process model, but also according to an intuitionist approach to moral judgment such as the ADC model (please see **line 677 to 697**).

*It seems rather problematic to characterise a scenario where the outcome is uncertain as less plausible; in real life the outcome is often uncertain, ie, the more uncertain the more realistic it is. Yet the authors say “In the implausible versions of the dilemma, the consequences of the utilitarian option were not certain”; to me, that sounds very plausible.*

**Answer:** Thank you for your comment that "characterizing a scenario with an uncertain outcome as less plausible could be problematic".

The plausibility of the scenario and the plausibility of the utilitarian action are indeed two different aspects of realism as we explained in the introduction section of the manuscript (**line 284 to 324**). In experiment 2, it is indeed the plausibility of the utilitarian action (i.e., plausibility of stated consequences and plausibility of better alternative actions) and not the plausibility of the scenario (i.e., the possible occurrence of the story in reality) that has been manipulated. Thus, this factor is named "plausibility of the action" with the two modalities "plausible vs. implausible". To remove any ambiguity throughout the manuscript, we have therefore replaced the denomination "plausible/implausible versions" by "versions with plausible/implausible action").

Your comment that “"characterizing a scenario with an uncertain outcome as less plausible could be problematic" nevertheless led us to consider the hypothesis that the plausibility of the scenario and the plausibility of the action might be related. We agree with your reasoning that, because real-world problems often involve little certainty about the outcome of the action, presenting the consequences of utilitarian action as certain might actually make the scenario less plausible. Consistent with this hypothesis, we should have observed a significant main effect of plausibility of action on the perceived realism of the scenario. However, analyses of variance (ANOVAs) conducted on the three dimensions of perceived realism showed that perceived realism was not influenced by Plausibility of action. Specifically, versions of the dilemma with a plausible action were perceived as equally plausible, factual, and typical as the others, regardless of the Type of dilemma (Contextualized vs. non contextualized within the Covid-19 pandemic). These results therefore rule out, at least in our experiment, the hypothesis of a link between the plausibility of action and the plausibility of the scenario. These results were added to the manuscript (**Line 573 to 578**). In general, measuring, as we do, the perceived realism of scenarios and the plausibility of the action in sacrificial dilemmas appears to be a necessity for a better understanding of moral responses.

*Why does the “plausible” version of the Covid-19 scenario in study 2 say “a virus causing respiratory irritation” rather than stating that it’s Covid-19?
The only difference between the plausible and implausible versions is the added explanation at the end. To many people that part would be obvious, so it’s not clear how it can be used to differentiate based on plausibility.*

**Answer:** Thank you for your careful reading of the manuscript. It is indeed an error that probably occurred during the translation of the manuscript. In their original version (in French), the plausible action version differed from the implausible action version only by the added information related to the plausibility of the action. The correct version of the scenario is the one described in the Study 1 material. The error you pointed out has been corrected in the material section of Study 2 (please **see Table 4**).

*Also, it’s extremely implausible to say that “this solution guarantees a 100% probability of saving them” – there’s never a 100% probability of saving any patient, much less one who needs oxygen on the intensive care unit, where around half of patients die.*

*The same problem applies to the plausible version of the antibiotics example – it remains extremely implausible and the explanation that’s designed to increase plausibilty actually descreases it by making implausible statements. However, the antibiotic example because, while denying oxygen will almost certainly kill a patient, providing antibiotics only increases chances of survival.*

**Answer:** We agree with you that nothing is ever certain, in the sense of "100% guaranteed". But as you point out previously the plausibility of utilitarian action has been underestimated in research on moral judgment. Your comparison of the trolley scenario (impersonal dilemma) and the footbridge scenario (personal dilemma) helps us to understand how this factor can explain the greater number of utilitarian responses in the former than in the latter. In accordance with your reasoning, Shou et al. (2020) showed that participants generally had greater certainty that the five people would survive if they decided to kill the individual in impersonal dilemmas compared to personal dilemmas.

In these dilemmas, as in the triage dilemma used in our experiments, it would indeed be a mistake to assume that participants interpret scenario outcomes as certain. Providing outcome certainty information in the form of probabilistic (100%) information allows the outcome to be perceived as more certain compared to scenarios that do not provide this information, but it also reduces the variability between participants' ratings of the certainty of the dilemma's outcome. In line with recent research on moral judgment that manipulates and controls for the degree of certainty of perceived outcomes (Shou et al., 2020, Körner et al., 2019), we used numerical probability (accurate and reliable information). To highlight that the manipulation of plausibility of action was consistent with previous work, we inserted additional elements in the material section of experiment 2 (see **line 519-528**).

In our study, the manipulation check confirmed that the stated consequences were judged to be more certain in the versions of the dilemma containing information about the certainty of the outcomes, compared to the ones without such information.

Consistent with results obtained with hypothetical dilemmas (Shou et al., 2020, Körner et al., 2019), our results showed that moral choices were significantly associated with beliefs about the probabilities of outcomes in real-word dilemmas such as the triage dilemma.

Finally, the fact that plausibility of action did not significantly influence participants' moral judgment underscores the necessity of distinguishing judgment and choice of action in moral responses.

*Telling participants that one option guarantees 100% that five lives will be saved clearly reduces real-world plausibility, but increases certainty and makes the choice easier*

*Given all these issues, I’m not sure how valid any of the results are.*

**Answer:** We partly agree with you that “Telling participants that one option guarantees 100% that five lives will (…) increases certainty and makes the choice easier”. However, our results do not sustain the idea that this manipulation “reduces real-world plausibility of the dilemmas”. As we previously mentioned, the manipulation check confirmed that the stated consequences were perceived as more certain in the versions of the dilemma containing the probability information, probably helping participants to take the utilitarian option without interfering with the perceived realism of the scenario.

Your comment helps us to notice that this measure (i.e., perceived realism) is one of the strengths of our studies: this precaution allowed us to assess whether the versions contextualized within Covid-19 were indeed perceived as more realistic than versions non-contextualized within Covid-19. Secondly, it enabled us to show that perceived realism did not influence moral responses. Thirdly, distinguishing the perceived realism of the scenario from the plausibility of the action contribute to overcome the drawback of previous studies conducted on moral judgment.

These results offer the opportunity to reconsider criticisms made about hypothetical dilemmas: instead of focusing on the lack of realism (a factor that does not seem to influence moral responses), the certainty of action (a determinant of moral choices) deserves to be more thoroughly considered in moral dilemma studies.

This is closely linked to an idea mentioned by Dubljevića and Racine (2014): “In the case of hypothetical dilemmas, uncertainty related to the response options is silenced” (p.12). In that case, participants may therefore infer their own probabilities concerning certainty of outcomes. To solve this problem (i.e., the subjective ratings of the certainty), Shou et al. (2020) suggested controlling or measuring subjective judgments regarding dilemma outcomes. This additional information has been inserted in the conclusion of the revised manuscript (please see conclusion section, **line 738 to line 747**)

The certainty of outcomes is also one major component of the Agent Deed Consequence (ADC) model (Dubljevic & Racine, 2014, 2017). According to this model, the emergence of moral responses relies on the evaluation of three intuitive components: the agent (the person who is doing something), the action in question, and the consequence (or outcome) of the action. Unfortunately, this intuitionist approach of moral judgment was missing in the first version of the manuscript. It is now included in the revised version since it offers an alternative explanation of our results: in real-world dilemmas, such as the triage dilemma, moral decisions could be guided by these three intuitive components. Thus, this model signals the necessity to consider the relative weight individuals attribute to these three components while making moral decisions (please see discussion section, **line 677 to line 697**).

*“On the other hand, they might choose to act or not to act mainly according to the outcomes they could reasonably expect (i.e., “saving a maximum number of lives”), rather than following their moral considerations”.*

*Once again, this implies that utilitarian principles don’t count as moral considerations. A utilitarian would regarded maximising lives saved as the moral and ethical thing to do. The fact that a deontologist would disagree doesn’t mean that the utilitarian is wrong or unethical.*

**Answer:** We agree with you that the utilitarian principles should not be presented as unethical/ opposite to ethics. Indeed, a utilitarian would consider that maximizing lives saved is the moral and ethical thing to do. To take your comment into account, we also modified the corresponding passage: “On the other hand, they might choose to act or not to act mainly according to the outcomes they could reasonably expect (i.e., “saving a maximum number of lives”)” (see **line 664 to line 666**).

*“Our results reveal that if taking [the oxygen / antibiotics] of one patient saves with certainty the five and if there is no other way (via alternative actions) to save them, people tend to be more utilitarian in their choice of action than when the consequences are uncertain and alternative actions exist.”*

*Of course they do – but certainty is deeply implausible, which is problematic for this study.*

**Answer:** As previously mentioned, we revised the label of our variables to clarify that plausibility of action (plausible action version vs. implausible action version) should be distinguished from the realism of scenario. These are two distinct variables examined in the present study. Please keep in mind that, in our work, the results showed that the perceived realism of the scenario was not influenced by the Plausibility of action (as stated **line 573 to 578**).

**Reviewer #2:**

*These authors have conducted two experiments to explore factors that may affect hypothetical moral responses and actions. With regard to their finding that the respondents view the COVID-19 scenario as more realistic than the antibiotic scenario, it seems quite clear that the difference is attributable to the current pandemic. From my perspective as a clinician ethicist, those two scenarios are ethically indistinguishable.*

**Answer:** Thanks for pointing out this characteristic that the two versions of the triage dilemma also have in common. This is indeed important to signal to the readers that regarding ethical principles, the two versions are indistinguishable. This information has been added in the material section (**line 355 to 358**).

*It is interesting that people tended to say that they would be more likely to act than to say that action was morally appropriate. The impact of certainty of the ability of action to save more people makes sense and adds support to existing literature.*

**Answer:** We agree with you that one contribution of the present study is to confirm previous results obtained by Tassy et al. (2013) who showed that people are more utilitarian in their choice of action than in their judgment. As you pointed out, we extend their results by stressing the importance of plausibility of action on choice of action but not on moral judgment. We hope that this strength of our work is made salient in the revised version of the manuscript.

*I found the authors' decision to divide the methods, results, discussion into two parts to be quite odd. They should unite them into one.*

**Answer:** We are not sure to understand why two studies have to be presented as one. The way we presented the two studies corresponds to what we did; the two studies were not conducted simultaneously but successively, with the objective, in Study 2, of replicating the results obtained in Study 1 (i.e., no significant effect of perceived realism on moral responses), and of furthering the question of the impact of the plausibility of utilitarian action.

In our view, there are two advantages in presenting the two studies separately: to respect the real context in which the two studies were conducted, and to attempt to replicate the results to overcome the reproducibility crisis in scientific studies.

Nevertheless, your comment drew our attention to the fact that the material section of Study 2 was too long and redundant. Therefore, we have presented in this section only the two new versions of the Triage dilemma (i.e., the versions containing information about the plausibility of sacrificial action) since the other two versions were identical to those in Study 1. Thus, we reduced the material section of Study 2 while providing enough detail to allow qualified investigators to fully replicate our study.

*I am confused about one thing -- you said that consent is not required but you obtained it from the students. that seems desirable but inconsistent.*

**Answer:** As is usually required prior to any participation in an experiment, participants receive a consent form in which they attest that they are voluntarily taking part in the study, and that they can interrupt their participation whenever they wish. However, since the data are anonymous and the protocol does not present any major risk for individuals, validation by an ethics committee is not mandatory (please see **line 367-368**).
