# Supplementary material for: Contextualizing sacrificial dilemmas within Covid-19 for the study of moral judgment
Source: PLoS One. 2022 Aug 22;17(8):e0273521. doi: 10.1371/journal.pone.0273521 (PMC9394814; doi:10.1371/journal.pone.0273521)
Supplement: Attachment — Submitted filename: ResponseReviewersJuly2022.docx [file pone.0273521.s003.docx]

**Response to reviewers**

**Reviewer’s comments**

I have re-checked this paper and I would advice to shorten the introduction by clearly introducing the topic, why it is relevant, what is the state of the art in the field and why this manuscript addresses that topic and with which purpose/objective.

Currently, the introduction is 13 pages and the reader gets lost about the focus, its relevance and ground in the literature.

Thank you for your comments that helped us to revise the manuscript. We agree that the introduction section was too long. As requested, we shortened and reorganized the introduction so that the way the manuscript now addresses the topic is made more salient. Three pages were eliminated from the introduction section.

Following your recommendations, we began by directly introducing the topic with the major criticism of dilemmas classically used to study moral judgment: their lack of realism (Bauman et al., 2014). This criticism calls into question the ecological validity of measured moral responses. In line with previous research that has attempted to address this criticism, the goal of the two studies presented in this article is to 1) propose more realistic sacrificial dilemmas (the triage dilemma by using the crisis context of Covid-19), but also to measure the realism of the scenarios used. Indeed, even if the literature proposes several solutions to improve this realism (solutions that we present and criticize in our state of the art), to our knowledge no study measures the perceived realism of the dilemmas used. It is thus impossible to determine the role of this factor on moral responses.

The contribution of our work is therefore to study moral responses in sacrificial dilemmas which are designed to be more realistic than those used so far, and also to control the perceived realism of the dilemmas studied in order to be able to draw scientific conclusions regarding the role of this factor on moral responses.

To this end, we propose a sequence of two experimental studies in which perceived realism is assessed in its different aspects: the realism of the scenario (i.e., its plausibility, factuality, and typicity) in Study 1, and the realism of the scenario associated with the plausibility of the sacrificial action in Study 2.

Finally, these studies contribute to shedding light on the question of the perceived realism of the dilemmas on two quite different aspects of moral response (Tassy et al., 2013): the judgment we make about a moral response (i.e., "is it morally acceptable?") and the intended action (i.e., the choice we would make if faced with a sacrificial dilemma). To the extent that, for obvious reasons, our participants could not actually act (i.e., could not sacrifice one person or let 5 persons die), measuring their intended action when faced with a sacrificial dilemma (contextualized within the Covid-19 pandemic or not) sheds light on the influence of perceived realism on choice of action.

**Reviewer’s comments**

For the other comments, I would better explain the methodology of the study and everything that can help understanding the results.

We agree that the elements provided in the manuscript relating to the exclusion criterion were insufficient. To remove any ambiguity on this subject, we added information in the method section:

Before conducting the study, it was clear to us that participants with a relative or close friend who had died of Covid-19 should be excluded. This exclusion criterion is based on Tassy et al. (2013)’s results: they showed that emotional or genetic proximity between participants and dilemma protagonists influences moral responses. Indeed, responses (and especially the choice of action) are less utilitarian when the potential victim (i.e., the one to be sacrificed) is a relative (e.g., a brother, a cousin, a friend) than when he/she is a stranger. Given the number of victims of the Covid-19 pandemic, the likelihood that participants had a relative or close friend who died of Covid-19 had to be considered. Of the 489 participants, 29 were excluded on the basis of this criterion. This allowed us to avoid any bias coming from closeness of relationship of the decision-maker with the potential victim, a factor determining moral responses.

We decided not to ask the question “have you lost a close relative or friend to COVID-19” at the beginning of the experiment for the following reason: Asking the question at the beginning would have raised the saliency of the Covid-19 pandemic context. It was indeed risky to ask this question at the beginning of the experiment for an obvious reason: in the condition with the non-contextualized version of the TRIAGE dilemma, participants were not expected to make the connection with the Covid-19 pandemic. In other words, raising the saliency of the covid-19 pandemic context could have interfered with the context variable we manipulated in this study (dilemmas contextualized and non-contextualized within Covid-19) and introduced a bias.

Of the 489 participants, 29 participants (15 in Study 1 and 14 in Study 2) were excluded on the basis of this criterion. All participants were volunteers and those who were finally excluded from the sample were not informed that their contribution to this study was not taken into account due to this exclusion criterion.

**Reviewer’s comments**

As for the English, once the paper has been revised I suggest rechecking the wording.

A native English speaker rechecked the wording.
